# Artificial neural network based on strong track and square root UKF for INS/GNSS intelligence integrated system during GPS outage

**DOI:** 10.1038/s41598-024-64918-4

**Published:** 2024-06-17

**Authors:** Yi Yang, Xueyao Wang, Nan Zhang, Zhaohui Gao, Yingliang Li

**Affiliations:** School of Electronic Engineering, Xi’an Shiyou University, Xi’an, 710065 China

**Keywords:** INS/GNSS intelligence integrated system, Neural network learning, Square root UKF, Strong tracking UKF, Tracking coefficient, Aerospace engineering, Information technology

## Abstract

When INS/GNSS (inertial navigation system/global navigation satellite system) integrated system is applied, it will be affected by the insufficient number of visible satellites, and even the satellite signal will be lost completely. At this time, the positioning error of INS accumulates with time, and the navigation accuracy decreases rapidly. Therefore, in order to improve the performance of INS/GNSS integration during the satellite signals interruption, a novel learning algorithm for neural network has been presented and used for intelligence integrated system in this article. First of all, determine the input and output of neural network for intelligent integrated system and a nonlinear model for weighs updating during neural network learning has been established. Then, the neural network learning based on strong tracking and square root UKF (unscented Kalman filter) is proposed for iterations of the nonlinear model. In this algorithm, the square root of the state covariance matrix is used to replace the covariance matrix in the classical UKF to avoid the filter divergence caused by the negative definite state covariance matrix. Meanwhile, the strong tracking coefficient is introduced to adjust the filter gain in real-time and improve the tracking capability to mutation state. Finally, an improved calculation method of strong tracking coefficient is presented to reduce the computational complexity in this algorithm. The results of the simulation test and the field-positioning data show that the proposed learning algorithm could improve the calculation stability and robustness of neural network. Therefore, the error accumulation of INS/GNSS integration is effectively compensated, and then the positioning accuracy of INS/GNSS intelligence integrated system has been improved.

## Introduction

With the rapid development of science and technology, there are many kinds of navigation systems used in aviation, aerospace, voyage and ground vehicles. Single navigation system has its own advantages and disadvantages, and it is difficult to meet the needs of long-term and high-performance navigation. Therefore, the navigation system is developing in the direction of integration. Compared with the single navigation system, the integrated navigation system has stronger cooperation transcendence function, redundancy complementary function, wider application range and stronger reliability^1,2^.

Inertial navigation system (INS) and global navigation satellite system (GNSS) are two kinds of commonly used navigation instrument. They have their own advantages and disadvantages in application. The former has strong autonomy, high short-time accuracy and continuous output, but the positioning error accumulates with time^3–6^; the latter has high positioning and speed measurement accuracy, and the positioning error does not accumulate with time, but the output information is discontinuous and vulnerable to interference^7–9^. The combination of these two instrument can achieve complementary advantages, which can significantly improve the comprehensive performance of the navigation system. At present, INS/GNSS integrated system has been widely used in aviation, aerospace, vehicle navigation and other fields. It is an ideal integrated navigation system^10–12^.

However, the performance of INS/GNSS integration will be affected in the case of insufficient number of visible satellites in application. For example, in the narrow area with dense high-rise buildings, reservoirs in deep mountains and canyons, underground tunnels and buildings with poor observation environment, the positioning accuracy is greatly decreased due to the insufficient number of visible satellites, and even the GNSS can not conduct positioning due to "loss" of signal completely^13,14^. At this time, the position error of INS will accumulate with time, and the position accuracy of INS/GNSS integration system will decline rapidly. Therefore, improving the performance of INS when it works independently is of great significance in the application.

Artificial neural network (ANN) is widely used in the modeling and optimization of INS/GNSS integrated system because of its strong nonlinear approximation ability, which is suitable for large-scale, parallel mode and complex or unknown mechanism problems. Aboelmagd et al. suggests the use of input-delayed neural network to model both position and velocity errors of INS based on current and some past navigation samples^13^. This research results in a more reliable positioning solution during long GPS outages. However, the traditional neural network method still has the following defects when applied to INS/GNSS integration. On the one hand, when the GNSS is available, the back propagation based neural network (BP-NN) is inefficient and this process depends on the the accuracy of learning samples heavily. On the other hand, the error estimation ability of neural network model will be affected by the accuracy of weight updating during the satellite signal interruption. In other words, the robustness of classical BP-NN is poor^15,16^.

In order to overcome the shortcomings above, Tan employed gradient optimization technology to adjust the learning step size of BP algorithm adaptive^17^. The test results show that the proposed algorithm could improve the efficiency of weight updating and reduces the steady-state error. However, this algorithm is still a local search based update method in essence. When the research objective is a complex nonlinear function, it is easy to fall into local extreme. Yang et al. combined genetic algorithm (GA) with fuzzy neural network to solve the problem that initial training parameters of the FNN model generate randomly. Simulation experiments verifies that the proposed FNN-GA control strategy is effective and reliable^18^. However, GA does not have the ability to fine tune in the local search space, and it is difficult to balance the selection of GA parameters and the convergence speed of the algorithm.

In order to further improve the overall performance of NN, the nonlinear filter technology, such as extended Kalman filter (EKF) and unscented Kalman filter (UKF), were used to neural network learning in recent years^19,20^. The premise of applying this technology is to establish a nonlinear system model with weight. This update mechanism is completely different from BP-NN based on local search. Compared with extended Kalman filter based neural network (EKF-NN), unscented Kalman filter neural network (UKF-NN) does not need to calculate Jacobi matrix and linearize the model, and it is easy to realize the optimal estimation of nonlinear system^21^. Therefore, UKF-NN has attracted more attention and has been applied in various fields. For instance, Ma et al. proposed a global information fusion model based on UKF-NN for ship navigation system, and the test results show that this method is effective and feasible^22^.

However, UKF-NN has also defects in application. The performance of UKF-NN mainly depends on UKF, so UKF-NN can not avoid the inherent defects of UKF, which results in the following two issues in application.

1. In the process of iterative calculation for covariance matrix, UKF-NN is easy to cause numerical instability due to rounding error and noise, resulting in the loss of symmetry and positive definiteness of the state covariance matrix, and then making the weight updating invalid^23–25^.
2. The filter gain of classical UKF can not be adjusted real-time, so it lacks the ability to track environmental changes^26,27^. At this time, once the samples for weight updating are disturbed by mutation, the stability and robustness of neural network based error estimation model will be greatly affected, so as to reduce the position accuracy of INS/GNSS integration.

Based on the above analysis with the related work, an novel neural network learning algorithm based on strong tracking and square root UKF will be studied for INS/GNSS intelligent integrated system. This algorithm combines the self-adjusting of strong tracking filter and the numerical stability of square root filter. Thus it could further improve the accuracy and real-time of neural network learning, and then enhance the robustness of neural network model, so as to solve the problem that the INS/GNSS integration is unavailable while the satellites are interrupted. The experiment tests were carried out by laboratory simulated data and the field-positioning data of a vehicle navigation in Xi’an, China, which verified the effectiveness and accuracy of the proposed neural network learning algorithm for INS/GNSS intelligent integrated system.

## The improved strong tracking and square root UKF based neural network

### The nonlinear system model for neural network learning

**Figure 1:**
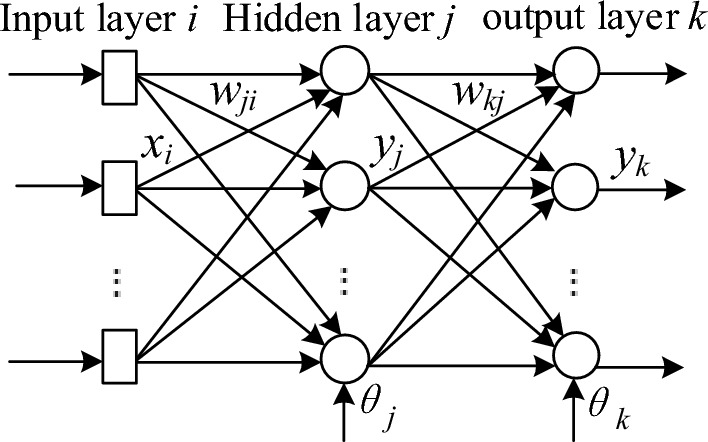
The structure of three layers feed-forward neural networks.

As shown in Fig. 1, a typical feed-forward neural network is composed of input layer, hidden layer and output layer. The input layer is composed of linear neurons, and the hidden layer and output layer neurons are hyperbolic tangent functions.

Therefore, the transmission relationship of a three-layer feed-forward neural network is as follow.

1 $$y_{j} = f\left( {\sum\limits_{i = 1}^{I} {w_{ji} x_{i} + \theta_{j} } } \right)$$

2 $$y_{k} = f\left( {\sum\limits_{j = 1}^{J} {w_{kj} y_{j} + \theta_{k} } } \right)$$

where *x*_*i*_ is output of the *i*-th neuron in the input layer; *w*_*ji*_ is the weight between the *i*-th neuron in the input layer and the *j*-th neuron in the hidden layer; *θ*_*j*_ is the threshold of the *j*-th neuron in the hidden layer; *y*_*j*_ is the output of the *j*-th neuron in the hidden layer; *w*_*kj*_ is the weight between the *j*-th neuron in the hidden layer and the *k*-th neuron in the output layer; *θ*_*k*_ is the threshold of the k-th neuron in the output layer; *y*_*k*_ is the output of the *k*-th neuron in the output layer. *f*(·) represents hyperbolic tangent function, generally expressed as follow.

3 $$f(x) = \frac{{1 - e^{ - 2x} }}{{1 + e^{ - 2x} }}$$

**Figure 2:**
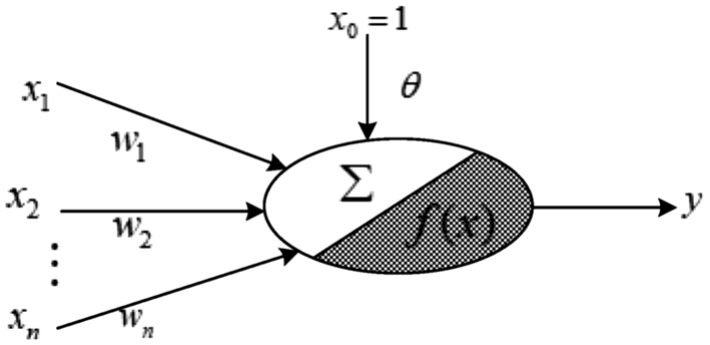
The nonlinear part of neuron structure.

It can be seen from the transmission relationship of the neurons above, neuron nodes are composed of weighted sum output *∑* and hyperbolic tangent function *f*(·), which is shown in Fig. 2. The former part is a linear function of the weight updating; the latter part is a nonlinear function of the weight updating.

For the nonlinear function in the shadow section of Fig. 2, the state space equation is established. Assume that the number of hidden layer nodes is *n* and the number of output layer nodes is *m*. Taking the weight matrix ***w*** ∈ ***R***^*n*×*m*^ between the hidden layer and the output layer as the state variable, and the expected output ***z*** ∈ ***R***^1×*m*^ of the network as the measured value, the nonlinear system model for neural network learning is as follow.

4 $$\left\{ {\begin{array}{*{20}c} {{\varvec{w}}_{{t + {1}/t}} = {\varvec{w}}_{t} } \\ {{\varvec{z}}_{{t + {1}/t}} = f({\varvec{w}}_{{t + {1}/t}} ,{\varvec{x}}_{{t + {1}}} ) + {\varvec{\theta}}_{t} } \\ \end{array} } \right.$$

where ***w***_*t*_ is the weight vector of the network output layer node at the *t*-th iteration; ***z***_*t*+1/*t*_ is the prediction output of the network at the *t*-th iteration; ***x***_*t*+1_ is the output of the hidden layer neurons at the (*t* + 1)-th iteration; ***θ***_*t*_ is the threshold vector of the neuron in the output layer at *t*-th iteration; *f*(·) express hyperbolic tangent function, which was given in Eq. (3).

### The strong tracking and square root UKF based neural network learning

#### The description of square root filter

The design of square root UKF is based on square root filter and standard UKF. Specifically, the square root form of the error covariance matrix is used instead in iterations. This method can ensure the non negative nature of the error covariance matrix and the numerical stability of the filtering algorithm. In addition, the process of updating Sigma points requires a large amount of computation, and performing Cholesky decomposition on the square root of the covariance matrix can effectively reduce computational complexity^26^. Therefore, square root filter can be used to solve the first issue mentioned in the previous section and can be executed according to the following two steps.

*Step 1* QR decomposition.

For any real matrix ***A***, it can be decomposed into the product of two matrices by QR decomposition, that is ***A*** = ***QR***, where ***Q*** is an orthogonal matrix and ***R*** is an upper triangular matrix. Its transpose matrix ***A***^T^ represents the return value of ***R*** in QR decomposition, denoted by *qr*(·). This step will be used in solving one-step predicted value of the square root matrix of the state/output covariance matrix.

*Step 2* Cholesky factor update.

According to matrix analysis theory, if the upper triangular matrix ***R*** is denoted as ***S***^T^, the matrix ***S*** can be regarded as the Cholesky decomposition of matrix ***P***, written as ***S*** = *chol*(***P***), where ***P*** = ***AA***^T^. Then, the Cholesky factor update of matrix ***P*** ± ***v***^½^***uu***^T^ can be written as ***S*** = *cholupdate*{***S***, ***u***, ± ***v***}, where ***u*** is usually a column vector. This step will be used in solving the estimated value of the square root matrix of the state/output covariance matrix.

#### The description of strong tracking filter

On the other hand, an tracking factor is introduced into standard UKF to adjust the size of the output prediction covariance matrix, and then the filter gain matrix will be adjusted online to enhance the algorithm's self-adaptability. However, the following two conditions should be met while the gain matrix is adjusted.

5 $$E\left[ {\left( {{\varvec{w}}_{k} - \hat{\user2{w}}_{k} } \right)\left( {{\varvec{w}}_{k} - \hat{\user2{w}}_{k} } \right)^{\text{T}} } \right] = min$$

6 $$E\left( {{\varvec{\varepsilon}}_{t} {\varvec{\varepsilon}}_{t + j}^{T} } \right) = 0,{\kern 1pt} {\kern 1pt} {\kern 1pt} {\kern 1pt} {\kern 1pt} t = {0,1,2,} \cdots {;}j = {1,2,} \cdots$$

where ***ε*** is the residual sequence of measured values.

It can be seen from Eq. (6) that the residual sequences ***ε*** must be orthogonal at any time. According to the principle of orthogonality, it is essentially to add a residual output sequence on the premise of the minimum variance performance index of state variable residual estimation. When the above equation is satisfied, the filter gain matrix can be adjusted in real time by adding strong tracking factor, and then the the purpose of tracking the actual system state will be achieved. Therefore, strong tracking filter can be used to solve the second issue mentioned in the previous section.

#### Design on strong tracking and square root UKF for neural networks

This section combines the strong tracking filter and square root filter on the basis of UKF-NN to obtain the strong tracking and square root UKF based neural network (STSR-NN). The computing steps for STSR-NN are as follows.

The nonlinear system equations for neural network learning are discretized as follows.

7 $$\left\{ \begin{gathered} {\varvec{w}}_{{t + {1}}} = {\varvec{w}}_{t} + {\varvec{\omega}}_{t} \hfill \\ {\varvec{z}}_{{t + {1}}} = f({\varvec{w}}_{t} ,{\varvec{x}}_{{t + {1}}} ,{\varvec{\theta}}_{t} ) + {\varvec{\nu}}_{t} \hfill \\ \end{gathered} \right.$$

where ***w***_*t*_ is the weight vector at *t*-th iteration; *ω*_*t*_ and *v*_*t*_ are the system noise and the measurement noise, respectively, meet the requirements of *ω*_*t*_ ~ *N*(0, ***Q***_*t*_), *v*_*t*_ ~ *N*(0, ***R***_*t*_); *f*(·) is the nonlinear measurement matrix, which was given in Eq. (4); ***z***_*t*+*1*_ and ***x***_*t*+*1*_ show the output and input vector of neural network, whose specific physical meaning will be given in Section IV; ***θ***_*t*_ is the threshold vector of hyperbolic tangent neurons at *t*-th iteration.

1. Initialization

The initial threshold bias of each neuron is set as any non-zero constant value, all connection weights of the network are small random numbers, and the initial variance matrix is a diagonal matrix.

(2) Computing the sigma points for the *t*-th iteration

8 $$\left\{ \begin{gathered} {\varvec{\chi}}_{t}^{0} = {\varvec{w}}_{t} \hfill \\ {\varvec{\chi}}_{t}^{i} = {\varvec{w}}_{t} + \gamma {\varvec{S}}_{t} {\kern 1pt} {\kern 1pt} {\kern 1pt} {\kern 1pt} {\kern 1pt} {\kern 1pt} {\kern 1pt} {\kern 1pt} {\kern 1pt} {\kern 1pt} {\kern 1pt} {\kern 1pt} i = 1,2,...,n \hfill \\ {\varvec{\chi}}_{t}^{i} = {\varvec{w}}_{t} - \gamma {\varvec{S}}_{t} {\kern 1pt} {\kern 1pt} {\kern 1pt} {\kern 1pt} {\kern 1pt} {\kern 1pt} {\kern 1pt} {\kern 1pt} {\kern 1pt} {\kern 1pt} {\kern 1pt} {\kern 1pt} i = n + 1,n + 2,...,2n \hfill \\ \end{gathered} \right.$$

where ***χ*** are the sigma points; ***S*** is the square root matrix of the state covariance matrix in UKF. The parameter *γ* is represented as follow.

9 $$\gamma = \sqrt {n + b}$$

where parameter *n* is the dimension of the nonlinear system in Eq. (7), which is also the number of hidden layer neurons for neural network learning. The parameter *b* is represented as follow.

10 $$b = \alpha^{2} \left( {n + K} \right) - n$$

where parameter *α* is the scale factor, which is generally set between 0.001 and 1; *K* is the third scale factor, which is usual set to 0.

(3) Prediction Process

11 $${\varvec{\chi}}_{{t + {1}/t}}^{i} = {\varvec{\chi}}_{t}^{i}$$

where ${\varvec{\chi}}_{{t + {1}/t}}^{i}$ is the one-step predicted value of the sigma point *i* in (*t* + 1)-th iteration.

12 $${\varvec{w}}_{{t + {1/}t}} = \sum\limits_{i = 0}^{2n} {W_{i}^{m} } {\varvec{\chi}}_{{t + {1/}t}}^{i}$$

where ***w***_*t*+1/*t*_ is the one-step predicted value of weight vector at (*t* + 1)-th iteration and the weight coefficient $W_{i}^{m}$ is given as follow.

13 $$W_{i}^{m} = \left\{ \begin{gathered} \frac{\lambda }{(n + \lambda )},{\kern 1pt} {\kern 1pt} {\kern 1pt} {\kern 1pt} {\kern 1pt} {\kern 1pt} {\kern 1pt} {\kern 1pt} {\kern 1pt} {\kern 1pt} {\kern 1pt} {\kern 1pt} i = 0 \hfill \\ \frac{1}{2(n + \lambda )},{\kern 1pt} {\kern 1pt} {\kern 1pt} {\kern 1pt} {\kern 1pt} i \ne 0 \hfill \\ \end{gathered} \right.$$

where *λ* is the tracking coefficient, whose computing method will be given in step (5).

14 $$\hat{\user2{S}}_{{t + {1}/t}} = qr\left[ {\sqrt {W_{i}^{c} } \left( {{\varvec{\chi}}_{{t + {1}/t}}^{{{1:}2n}} - {\varvec{w}}_{{t + {1}/t}} } \right),\sqrt {{\varvec{Q}}_{t} } } \right]$$

where the symbol *qr* is represented the QR decomposition calculating in square root UKF, $\hat{\user2{S}}_{t + 1/t}$ is the one-step predicted value of the square root matrix of the state covariance matrix and ${\varvec{Q}}_{t}$ is the variance of the system noise in *t*-th iteration. The weight coefficient $W_{i}^{c}$ is given as follow.

15 $$W_{i}^{c} = \left\{ \begin{gathered} \frac{\lambda }{(n + \lambda )} + (1 + \beta - \alpha^{2} ),{\kern 1pt} {\kern 1pt} {\kern 1pt} {\kern 1pt} {\kern 1pt} {\kern 1pt} {\kern 1pt} {\kern 1pt} {\kern 1pt} {\kern 1pt} {\kern 1pt} {\kern 1pt} i = 0 \hfill \\ \frac{1}{2(n + \lambda )},{\kern 1pt} {\kern 1pt} {\kern 1pt} {\kern 1pt} {\kern 1pt} {\kern 1pt} {\kern 1pt} {\kern 1pt} {\kern 1pt} {\kern 1pt} {\kern 1pt} {\kern 1pt} {\kern 1pt} {\kern 1pt} {\kern 1pt} {\kern 1pt} {\kern 1pt} {\kern 1pt} {\kern 1pt} {\kern 1pt} {\kern 1pt} {\kern 1pt} {\kern 1pt} {\kern 1pt} {\kern 1pt} {\kern 1pt} {\kern 1pt} {\kern 1pt} {\kern 1pt} {\kern 1pt} {\kern 1pt} {\kern 1pt} {\kern 1pt} {\kern 1pt} {\kern 1pt} {\kern 1pt} {\kern 1pt} {\kern 1pt} {\kern 1pt} {\kern 1pt} {\kern 1pt} {\kern 1pt} {\kern 1pt} {\kern 1pt} {\kern 1pt} {\kern 1pt} {\kern 1pt} {\kern 1pt} {\kern 1pt} {\kern 1pt} {\kern 1pt} {\kern 1pt} {\kern 1pt} {\kern 1pt} {\kern 1pt} {\kern 1pt} {\kern 1pt} {\kern 1pt} {\kern 1pt} {\kern 1pt} {\kern 1pt} {\kern 1pt} {\kern 1pt} {\kern 1pt} {\kern 1pt} {\kern 1pt} {\kern 1pt} {\kern 1pt} {\kern 1pt} {\kern 1pt} {\kern 1pt} i \ne 0 \hfill \\ \end{gathered} \right.$$

where *β* is the adjusted parameter and usually set as a positive number. The remaining parameters are as above.

16 $${\varvec{S}}_{{t + {1}/t}} = chol\left( {\hat{\user2{S}}_{{t + {1}/t}} ,{\varvec{\chi}}_{{t + {1}/t}}^{0} - {\varvec{w}}_{{t + {1}/t}} ,W_{0}^{c} } \right)$$

where ${\varvec{S}}_{t + 1/t}$ is the estimated value of the square root matrix of the state covariance matrix in (*t* + 1)-th iteration and the symbol *chol* is represented the Cholesky factor updating in square root UKF.

17 $${\varvec{\zeta}}_{{t + {1}/t}}^{i} = f\left( {{\varvec{\chi}}_{{t + {1}/t}}^{i} } \right)$$

where ${\varvec{\zeta}}_{t + 1/t}^{i}$ is the nonlinear output of ${\varvec{\chi}}_{{t + {1}/t}}^{i}$.

18 $${\varvec{z}}_{{t + {1}/t}} = \sum\limits_{i = 0}^{2n} {W_{i}^{m} } {\varvec{\zeta}}_{{t + {1}/t}}^{i}$$

where ${\varvec{z}}_{t + 1/t}^{{}}$ is the estimated value of output of neural network in (*t* + 1)-th iteration.

(4) Renewal Process

19 $${\varvec{P}}_{{t + {1}/t}}^{(XZ)} = \sum\limits_{i = 0}^{2n} {W_{i}^{c} \left[ {{\varvec{\chi}}_{{t + {1}/t}}^{i} { - }{\varvec{w}}_{{t + {1}/t}} } \right]} \left[ {{\varvec{\zeta}}_{{t + {1}/t}}^{i} \user2{ - z}_{{t + {1}/t}} } \right]^{T}$$

where ${\varvec{P}}_{t + 1/t}^{(XZ)}$ is the cross covariance matrix in (*t* + 1)-th iteration.

20 $$\hat{\user2{S}}_{{t + {1}/t}}^{Z} = qr\left[ {\sqrt {W_{i}^{c} } \left( {{\varvec{\zeta}}_{{t + {1}/t}}^{{{1:}2n}} - {\varvec{z}}_{{t + {1}/t}} } \right),\sqrt {{\varvec{R}}_{t} } } \right]$$

where $\hat{\user2{S}}_{t + 1/t}^{Z}$ is the one-step predictive of the square root of the output covariance matrix in (*t* + 1)-th iteration and ${\varvec{R}}_{t}$ is the variance of the measurement noise in *t*-th iteration.

21 $${\varvec{S}}_{{t + {1}/t}}^{Z} = \lambda_{t + 1} \cdot chol\left( {\hat{\varvec{S}}_{{t + {1}/t}}^{Z},\, \zeta_{{t + {1}/t}}^{0} - {\varvec{z}}_{{t + {1}/t}},\, W_{0}^{c} } \right)$$

where ${\varvec{S}}_{t + 1/t}^{Z}$ is the estimated value of the square root of the output covariance matrix in (*t* + 1)-th iteration and *λ*_*t*+1_ is the tracking coefficient in (*t* + 1)-th iteration.

(5) Computing the Tracking Coefficient

At present, the traditional computing method for tracking coefficient is as follows.

22 $$\lambda_{t + 1} = \max \left\{ {1,\frac{{tr({\varvec{N}}_{t + 1} )}}{{tr({\varvec{M}}_{t + 1} )}}} \right\}$$

where *tr*(·) represents matrix tracing, and the matrix ***M***_*t*+1_ and ***N***_*t*+1_ are computing as follow.

23 $$\left\{ \begin{gathered} {\varvec{M}}_{t + 1} = {\varvec{H}}_{t + 1} {\varvec{F}}_{t + 1/t} {\varvec{P}}_{t + 1/t} {\varvec{F}}_{t + 1/t}^{T} {\varvec{H}}_{t + 1}^{T} \hfill \\ {\varvec{N}}_{t + 1} = {\varvec{C}}_{t + 1} - {\varvec{H}}_{t + 1} {\varvec{Q}}_{t + 1} {\varvec{H}}_{t + 1}^{T} - {\varvec{R}}_{t + 1} \hfill \\ \end{gathered} \right.$$

where

24 $${\varvec{P}}_{t + 1/t} = {\varvec{S}}_{t + 1/t} {\varvec{S}}_{t + 1/t}^{T}$$

25 $${\varvec{C}}_{t + 1} = \left\{ \begin{gathered} {\varvec{Y}}_{t + 1} {\varvec{Y}}_{t + 1}^{T} ,{\kern 1pt} {\kern 1pt} {\kern 1pt} {\kern 1pt} {\kern 1pt} {\kern 1pt} {\kern 1pt} {\kern 1pt} {\kern 1pt} {\kern 1pt} {\kern 1pt} {\kern 1pt} {\kern 1pt} {\kern 1pt} {\kern 1pt} {\kern 1pt} {\kern 1pt} {\kern 1pt} {\kern 1pt} {\kern 1pt} {\kern 1pt} {\kern 1pt} {\kern 1pt} {\kern 1pt} {\kern 1pt} {\kern 1pt} {\kern 1pt} {\kern 1pt} {\kern 1pt} {\kern 1pt} {\kern 1pt} {\kern 1pt} {\kern 1pt} {\kern 1pt} {\kern 1pt} {\kern 1pt} {\kern 1pt} {\kern 1pt} {\kern 1pt} {\kern 1pt} {\kern 1pt} {\kern 1pt} {\kern 1pt} {\kern 1pt} {\kern 1pt} {\kern 1pt} {\kern 1pt} {\kern 1pt} {\kern 1pt} {\kern 1pt} {\kern 1pt} {\kern 1pt} {\kern 1pt} {\kern 1pt} t = 0 \hfill \\ \frac{{\rho {\varvec{C}}_{t} + {\varvec{Y}}_{t + 1} {\varvec{Y}}_{t + 1}^{T} }}{1 + \rho },{\kern 1pt} {\kern 1pt} {\kern 1pt} {\kern 1pt} {\kern 1pt} {\kern 1pt} {\kern 1pt} {\kern 1pt} {\kern 1pt} {\kern 1pt} {\kern 1pt} {\kern 1pt} {\kern 1pt} {\kern 1pt} t \ge 1 \hfill \\ \end{gathered} \right.$$

26 $${\varvec{Y}}_{t + 1} = {\varvec{z}}_{t + 1} { - }{\varvec{H}}_{t + 1} {\varvec{w}}_{t + 1/t}$$

27 $${\varvec{F}}_{t + 1/t} = {\varvec{I}},\,{\varvec{H}}_{t + 1} = \frac{{\partial f({\varvec{w}}_{t + 1/t} )}}{{\partial {\varvec{w}}_{t + 1/t} }}$$

where *ρ* is the forgetting factor; ***F***_*t*+1/*t*_ and ***H***_*t*+1_ are the Jacobi expansion of the state equation and the measurement equation, respectively. For the research object of this article, ***F***_*t*+1/*t*_ could be set to a.

unit matrix.

(6) Computing the Gain Matrix

28 $${\varvec{K}}_{t + 1} = \left( {\frac{{{\varvec{P}}_{t + 1/t}^{{{(}XZ{)}}} }}{{\left( {{\varvec{S}}_{t + 1/t}^{Z} } \right)^{T} }}} \right)\left( {{\varvec{S}}_{t + 1/t}^{Z} } \right)^{{{ - 1}}}$$

where ***K***_*t*+1_ is the gain matrix in (*t* + 1)-th iteration.

(7) Weights Updating

29 $${\varvec{w}}_{{t + {1}}} = {\varvec{w}}_{{t + {1/}t}} + {\varvec{K}}_{t + 1} \left( {{\varvec{z}}_{t + 1} - {\varvec{z}}_{{t + {1/}t}} } \right)$$

30 $${\varvec{U}} = {\varvec{K}}_{t + 1} {\varvec{S}}_{t + 1/t}^{Z}$$

31 $${\varvec{S}}_{t + 1}^{{}} = chol\left( {{\varvec{S}}_{t + 1/t}^{{}} ,{\varvec{U}}, - 1} \right)$$

where ***w***_*t*+1_ and ***S***_*t*+1_ are the updated weight matrix and the square root of the output covariance matrix after (*t* + 1)-th iteration.

The neural network learning will be perform according to steps above until the output error of neural network meet the requirements.

### The improved computing method for strong tracking coefficient

According to the traditional computing method discussed in previous section, the Jacobian matrix needs to be computing in order to gain the tracking coefficient. For the measurement equation of weight updating, the computation of STSR-NN will be significantly increased when the dimension of weight matrix is high. In order to overcome this issues, an improved computing method for tracking coefficient is presented in this section, and thus the improved strong tracking and square root UKF based neural network (ISTSR-NN) will be established.

#### Theorem

It is assumed that the process matrix of the strong tracking UKF are represented as follow.

32 $${\varvec{P}}_{t + 1/t} = E\left[ {\left( {{\varvec{w}}_{t + 1} - {\varvec{w}}_{t + 1/t} } \right)\left( {{\varvec{w}}_{t + 1} - {\varvec{w}}_{t + 1/t} } \right)^{T} } \right] = {\varvec{P}}_{t} + {\varvec{Q}}_{t + 1}$$

33 $${\varvec{P}}_{t + 1/t}^{(ZZ)} = E\left[ {\left( {{\varvec{z}}_{t + 1} - {\varvec{z}}_{t + 1/t} } \right)\left( {{\varvec{z}}_{t + 1} - {\varvec{z}}_{t + 1/t} } \right)^{T} } \right] = {\varvec{H}}_{t + 1} {\varvec{P}}_{t + 1/t} {\varvec{H}}_{t + 1}^{T} + {\varvec{R}}_{t + 1}$$

34 $$\begin{gathered} {\varvec{P}}_{t + 1/t}^{(XZ)} = E\left[ {\left( {{\varvec{w}}_{t + 1} - {\varvec{w}}_{t + 1/t} } \right)\left( {{\varvec{z}}_{t + 1} - {\varvec{z}}_{t + 1/t} } \right)^{T} } \right]{\kern 1pt} = E\left[ {\left( {{\varvec{w}}_{t + 1} - {\varvec{w}}_{t + 1/t} } \right)\left( {{\varvec{H}}_{t + 1} \left( {{\varvec{w}}_{t + 1} - {\varvec{w}}_{t + 1/t} } \right)} \right)^{T} } \right] + E\left[ {\left( {{\varvec{w}}_{t + 1} - {\varvec{w}}_{t + 1/t} } \right){\varvec{\nu}}_{t + 1}^{T} } \right] \hfill \\ {\kern 1pt} {\kern 1pt} {\kern 1pt} {\kern 1pt} {\kern 1pt} {\kern 1pt} {\kern 1pt} {\kern 1pt} {\kern 1pt} {\kern 1pt} {\kern 1pt} {\kern 1pt} {\kern 1pt} {\kern 1pt} {\kern 1pt} {\kern 1pt} {\kern 1pt} {\kern 1pt} {\kern 1pt} {\kern 1pt} {\kern 1pt} {\kern 1pt} {\kern 1pt} {\kern 1pt} {\kern 1pt} {\kern 1pt} {\kern 1pt} {\kern 1pt} {\kern 1pt} = {\varvec{P}}_{t + 1/t} {\varvec{H}}_{t + 1}^{T} + E\left[ {\left( {{\varvec{w}}_{t + 1} - {\varvec{w}}_{t + 1/t} } \right){\varvec{\nu}}_{t + 1}^{T} } \right] \hfill \\ \end{gathered}$$

where ***P***_*t*+1/*t*_ is the state covariance matrix, ${\varvec{P}}_{t + 1/t}^{(ZZ)}$ is the output covariance matrix and ${\varvec{P}}_{t + 1/t}^{(XZ)}$ is the cross-covariance matrix. The remaining parameters are the same as discussed above.

Then, the matrix ***M***_*t*_ and ***N***_*t*_ to computing the tracking coefficient *λ* can be expressed as follow.

35 $${\varvec{N}}_{t + 1} = {\varvec{C}}_{t + 1} - \left( {{\varvec{P}}_{t + 1/t}^{(XZ)} } \right)^{T} \left( {{\varvec{S}}_{t + 1/t} {\varvec{S}}_{t + 1/t}^{T} } \right)^{ - 1} {\varvec{Q}}_{t + 1} \cdot \left( {{\varvec{S}}_{t + 1/t} {\varvec{S}}_{t + 1/t}^{T} } \right){\varvec{P}}_{t + 1/t}^{(XZ)} - {\varvec{R}}_{t + 1}$$

36 $${\varvec{M}}_{t + 1} = \left( {{\varvec{P}}_{t + 1/t}^{(XZ)} } \right)^{T} \left( {{\varvec{S}}_{t + 1/t} {\varvec{S}}_{t + 1/t}^{T} } \right)^{ - 1} \cdot \left( {{\varvec{S}}_{t + 1/t} {\varvec{S}}_{t + 1/t}^{T} - {\varvec{Q}}_{t + 1} } \right)\left( {{\varvec{S}}_{t + 1/t} {\varvec{S}}_{t + 1/t}^{T} } \right)^{ - 1} {\varvec{P}}_{t + 1/t}^{(XZ)}$$

where ***C***_*t*+1_ is computing according to Eq. (25).

#### Proof

Since the matrix ${(}{\varvec{w}}_{{t + {1}}} { - }{\varvec{w}}_{{t + {1/}t}} {)}$ and the noise matrix ***v***_*t*+1_ are orthogonal, the Eq. (34) could be rewritten as follow.

37 $${\varvec{P}}_{t + 1/t}^{(XZ)} = {\varvec{P}}_{t + 1/t} {\varvec{H}}_{t + 1}^{T}$$

Further, Eq. (32) could be rewritten as follow.

38 $${\varvec{P}}_{t} = {\varvec{P}}_{t + 1/t} - {\varvec{Q}}_{t + 1}$$

Suppose ***Q***_*t*+1_ is a positive definite matrix, and according to the UKF, the inverse matrix of the covariance matrix ***P***_*t*+1/*t*_ must exist, so Eq. (37) could be rewritten as follow.

39 $${\varvec{H}}_{t + 1}^{T} = \left( {{\varvec{P}}_{t + 1/t}^{{{(}XZ{)}}} } \right)^{T} \left( {{\varvec{P}}_{t + 1/t}^{T} } \right)^{{{ - 1}}}$$

The Eqs. (35) and (36) are established while the Eqs. (24), (38), and (39) are substituted into Eq. (23).

The **Theorem** has been proved.

Thus, the tracking coefficient *λ* could be computed by the Eqs. (22), (35), (36), (25), (26), and (39), (16) successively, and then the Jacobi expansion ***F***_*t*+1/*t*_ and ***H***_*t*+1_ of the state equation and the measurement equation are avoid to calculate. Furthermore, the ISTSR-NN has been presented.

## Design for intelligent integrated system based on ISTSR-NN

### The updating scheme for navigation information

The first step to establish an intelligent integrated system is to design an INS/GNSS integrated navigation scheme, which provides necessary training samples (navigation information) for neural networks.

**Figure 3:**
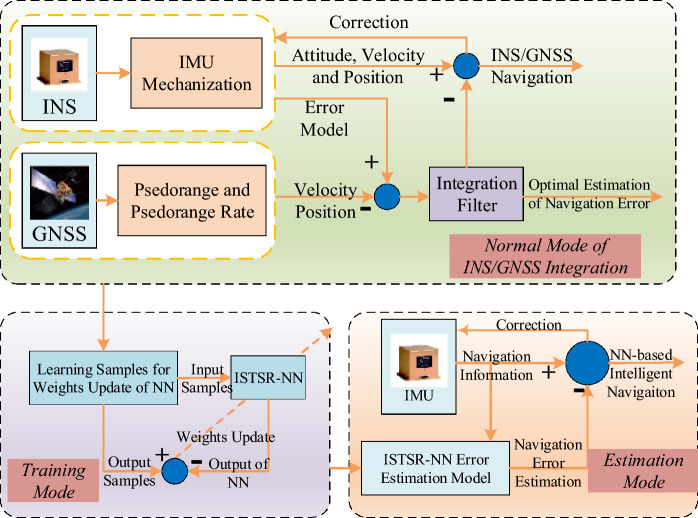
The diagram of intelligent navigation system based on ISTSR-NN.

As shown in Fig. 3, when the satellite signals are available, the difference between the navigation information (velocity and position) output by the GNSS receiver and INS are used as the measurement value, which are input into the integrated navigation filter to generate error estimation for INS. These error estimation can be used to correct INS after each measurement updating to improve the accuracy of INS. The navigation information (azimuth, velocity and position) output by INS are chosen as input samples for neural network training, while the INS error estimation output by the integration filter are chosen as output samples for neural network training. Therefore, a nonlinear mapping relationship was established between INS output and INS error estimation. Finally, the proposed training algorithm ISTSR in this paper is used to update the weights of the neural network.

As shown in Fig. 3, when the satellite signals are interrupted, the navigation information output by INS are still chosen as input samples to test the trained neural network model. This network model can simulate (predict) the INS error estimation output by the integrated navigation filter based on the knowledge and experience gained from learning. In other words, a trained neural network model can be used to replace the integrated navigation filter for correcting INS, and then improving the positioning accuracy when INS works alone.

### The structure design for ISTSR-NN

The second step of building the intelligent navigation system is to select the appropriate topology for ISTSR-NN. This includes the number of neurons in the input layer, hidden layer and output layer. In this paper, the optimal estimation of position error is selected as the output variable of the ISTSR-NN, which is shown in Eq. (40).

40 $$NN_{out} = \left[ {\delta {\varvec{P}}} \right]^{T} = \left[ {\begin{array}{*{20}c} {\delta L} & {\delta \lambda } \\ \end{array} } \right]^{T}$$

where *δL*, *δλ* are latitude, longitude errors after integration filter, respectively.

On the other hand, the velocity, position, attitude and its change rate in INS are selected as the input variable of the ISTSR-NN, which is shown in Eq. (41).

41 $$NN_{in} = \left[ {\begin{array}{*{20}c} {\Delta V_{E} } & {\begin{array}{*{20}c} {\Delta V_{N} } & {\Delta L} & {\Delta \lambda } & {\begin{array}{*{20}c} \phi & {\Delta \phi } \\ \end{array} } \\ \end{array} } \\ \end{array} } \right]^{T}$$

where *V*_*E*_ and *V*_*N*_ represent the velocity of the vehicle in the East and north directions respectively; *φ* represents the azimuth of the vehicle; and the prefix Δ represents the variation within a step length. Therefore, the number of input and output nodes for the intelligent navigation system are 6 and 2, respectively.

The most appropriate number of hidden neurons is application dependent and can only be decided empirically during the early stages of topology design^14^. It is very common that increase the number of hidden layer nodes from a smaller number gradually until the neural network performance reaches its optimal level. For the intelligent navigation system discussed in this article, the initial value of the number of hidden nodes will be set to 3, and then many different numbers of hidden neurons will be train until the best one selection.

## Performance evaluation and results

The INS/GNSS intelligent integrated system based on ISTSR-NN developed in this paper was tested through simulation and experiments, respectively. Comparison with BP-NN, UKF-NN, square root UKF based neural network (SR-NN), strong tracking UKF based neural network (ST-NN) and STSR-NN were conducted to evaluate the performance of the proposed ISTSR-NN comprehensively.

This paper uses these parameters to evaluate the performance of intelligence integrated system during GPS outage: root mean square error (RMSE), mean absolute error (MAE) scatter index (SI), and correlation coefficient (CC). These parameters are expressed as follows^28^.

42 $$RMSE = \sqrt {\frac{1}{n}\sum\limits_{i = 1}^{n} {\left( {y_{i} - \hat{y}_{i} } \right)^{2} } }$$

43 $$MAE = \frac{1}{n}\sum\limits_{i = 1}^{n} {\left| {y_{i} - \hat{y}_{i} } \right|}$$

44 $$SI = \sqrt {\frac{{\sum\limits_{i = 1}^{n} {\left[ {\left( {y_{i} - \overline{y}_{i}^{{}} } \right) - \left( {\hat{y}_{i} - \overline{\hat{y}}_{i}^{{}} } \right)} \right]^{2} } }}{{\sum\limits_{i = 1}^{n} {y_{i}^{2} } }}}$$

45 $$CC = \frac{{\sum\limits_{i = 1}^{n} {\left[ {\left( {y_{i} - \overline{y}_{i} } \right)\left( {\hat{y}_{i} - \overline{\hat{y}}_{i} } \right)} \right]} }}{{\sqrt {\sum\limits_{i = 1}^{n} {\left( {y_{i} - \overline{y}_{i} } \right)^{2} } \sum\limits_{i = 1}^{n} {\left( {\hat{y}_{i} - \overline{\hat{y}}_{i} } \right)^{2} } } }}$$

where $y_{i}$ and $\hat{y}_{i}$ are the *i*th target and predicted value, $\overline{y}_{i}$ and $\overline{\hat{y}}_{i}$ are the mean of target and predicted value, and *n* is the number of the data samples.

Moreover, to have a comprehensive evaluation of the proposed approach, the uncertainty interval U95 and the reliability percent of the NN models are calculated as follows^28^.

46 $$U95 = \left( {\frac{1.96}{n}} \right)\sqrt {\sum\limits_{i = 1}^{n} {\left( {y_{i} - \overline{y}_{i} } \right)^{2} + \sum\limits_{i = 1}^{n} {\left( {y_{i} - \hat{y}_{i} } \right)^{2} } } }$$

47 $$Reliability = \left( {\frac{100\% }{n}} \right)\sum\limits_{i = 1}^{n} {T_{i} }$$

where *T*_*i*_ is obtained in two steps. First, the relative average error (RAE) for each sample is calculated as follows.

48 $$RAE_{i} = \left| {\frac{{y_{i} - \hat{y}_{i} }}{{\hat{y}_{i} }}} \right|$$

Second, *T*_*i*_ = 1 if *RAE*_*i*_ ≤ Δ, otherwise, *T*_*i*_ = 0, where Δ is a threshold that determines the level of predicted error tolerability, which based on Chinese standards, is equal to 0.2^29^; so here also is set to 0.2.

### Simulation and analysis

**Figure 4:**
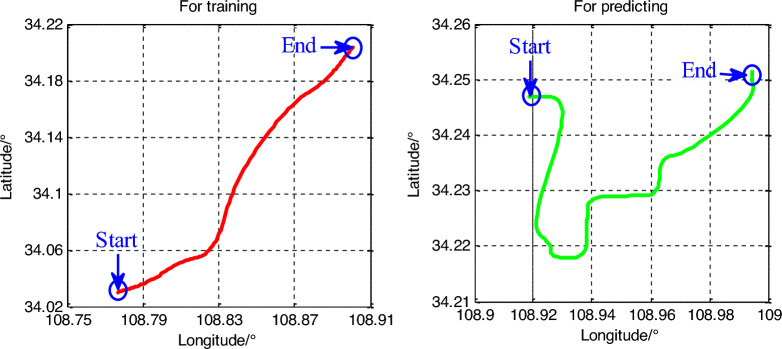
The movement trajectories of the vehicle.

Simulations were carried out to examine the performance of the proposed ISTSR-NN for vehicle positioning with INS/GNSS intelligent integrated system. Figure 4 depicts the dynamic movement trajectory of the vehicle, which was designed to involve various maneuvers such as linear motion, accelerated motion, uniform motion, turning and so on. The left one was used for neural networks training and the right one was used for predicting. The simulation parameters were described in Table 1.

**Table 1:** Simulation Parameters.

	Parameters		Values
Initial parameter	Initial position (longitude-latitude)		(108.902°, 34.205°)
	Initial velocity (east-north)		(5 m/s, 0 m/s)
	Initial azimuth		90°
Initial parameter errors	Initial position error (longitude-latitude)		(5 m, 5 m)
	Initial velocity error (east-north)		(0.1 m/s, 0.1 m/s)
	Initial azimuth error		0.5°
INS parameters	Gyro parameters	Constant drift	0.1°/h(1 $\sigma$)
		Measurement white noise	0.01°/h(1 $\sigma$)
	Accelerometer parameters	Zero-bias	5 × 10^-4^ g(1 $\sigma$)
		Measurement white noise	1 × 10^-4^ g(1 $\sigma$)
GPS parameters	Horizontal position error (RMS)		5 m
	Velocity error		0.5 m/s
	Data update rate		0.5 Hz
Simulation time	Training time: 2000s; Prediction time: 1200 s		

The simulation time for training was 2000s and the time for predicting was 1200 s. Moreover, the filtering period was 2 s and the parameters in ISTSR-NN were set to *K* = 0, *ρ* = 0.95, *α* = 0.05, *β* = 2. In order to accurately evaluate the performance of the proposed method, all the neural networks models are under the same simulation conditions.

#### Training performance evaluation

**Figure 5:**
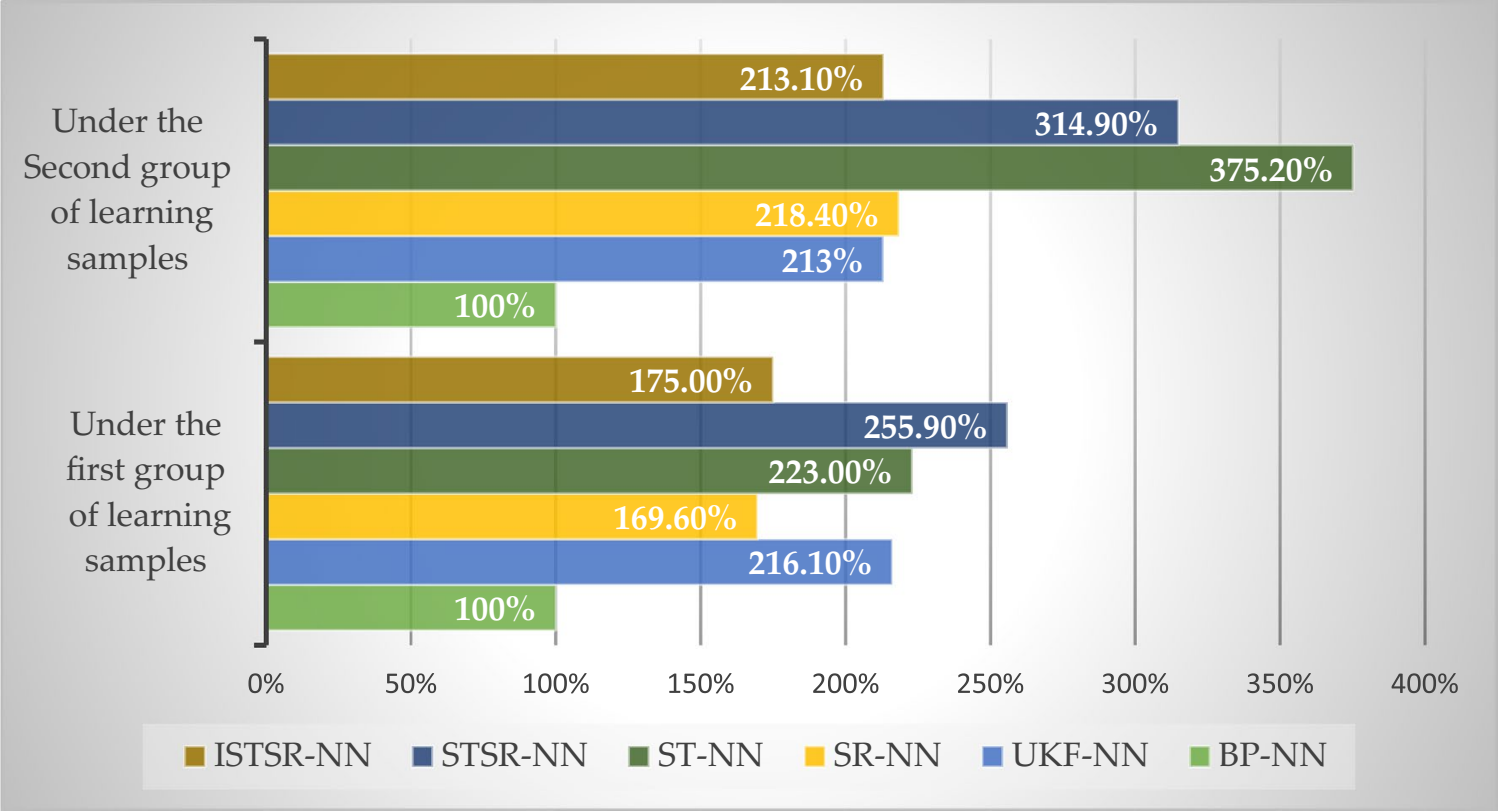
Relative computational efficiencies of BP-NN, UKF-NN, SR-NN, ST-NN, STSR-NN and ISTSR-NN.

The simulation tests were carried out on an Intel(R) Core(TM) i7-10700 2.9 GHz PC with 16 GB memory to study the computational efficiency of the proposed ISTSR-NN for two types of training samples in this section. The data from training trajectory was used as learning samples. The input and output of neural networks model are shown in Eqs. (40) and (41), and then update the network weights by BP-NN, UKF-NN, SR-NN, ST-NN, STSR-NN and ISTSR-NN, respectively. During the training process, the initial value of the number of hidden nodes will be set to 3, and then many different numbers of hidden neurons will be train until the best NN configuration decided, which are given in Table 2. As mentioned before, the hyperbolic tangent is used as the activation function. The training results are given in Table 2. Moreover, the computational efficiencies of the other learning algorithm relative to that of BP-NN are shown in Fig. 5.

**Table 2:** Statistics of learning process for neural network models.

Learning algorithms	Under the first sample group			Under the second sample group		
	Learning accuracy	Iterations (≤ 1000)	NN configuration	Learning accuracy	Iterations (≤ 1000)	NN configuration
BP-NN	0.001	513	6-14-2	0.01	467	6-17-2
UKF-NN	0.001	351	6-12-2	0.01	315	6-14-2
SR-NN	0.001	87	6-16-2	0.001	102	6-17-2
ST-NN	0.001	126	6-17-2	0.001	193	6-16-2
STSR-NN	0.001	58	6-14-2	0.001	65	6-14-2
ISTSR-NN	0.001	55	6-14-2	0.001	61	6-12-2

Table 2 and Fig. 5 show that when the actual output results of INS/GNSS combined filtering are used as the learning samples for the neural networks, the learning accuracy of all the six algorithms is 0.001. Under the same learning accuracy, BP-NN has the shortest computational time among all the models. UKF-NN’s computational time is 116.1% larger than that of BP-NN due to a vast number of calculations involved in the unscented transformation. The computational times of SR-NN and ST-NN are 69.6% and 123.0% larger than that of BP-NN due to the involvement of computing the square root matrix and the tracking coefficient, respectively. Further, since the two sub-filters are executed simultaneous, the computational time of STSR-NN is increased obviously comparing to those of SR-NN and ST-NN, whose computational time is 155.9% larger than that of BP-NN. In contrast, because of the advantage of the improved calculation method of the tracking coefficient, the computational time of ISTSR-NN is shorter than that of STSR-NN about 31.6%.

Moreover, although the additional computations were involved in Cholesky and QR decomposition during the learning process of SR-NN, the computational time of SR-NN is 21.5% shorter than that of UKF-NN. This is because the number of iterations required for SR-NN to achieve learning accuracy is only 87, which is much smaller than the 351 of UKF-NN. Therefore, it can be seen that square root filter does have better computational stability in the learning process of neural networks and thus requires fewer computational time to complete the learning process. A similar situation also occurs between ST-NN and ISTSR-NN. The extra calculations were involved in square root filter during the learning process of ISTSR-NN. However, the computational time of ISTSR-NN is 21.6% shorter than that of UKF-NN. This result proves that square root filtering can improve the computational stability of neural networks learning effectively once again.

On the other hand, when the second group of learning samples, with 10% random noise, are used for neural networks, the learning accuracy of BP-NN and UKF-NN could only reach 0.01. So the BP and UKF algorithm have poor fault tolerance for learning samples. The decrease in learning accuracy will directly affect the generalization ability of neural network models, leading to poor positioning accuracy of intelligent navigation systems based on BPNN and UKFNN. Although the learning accuracy of the other four neural network models are still 0.001, the number of iterations required have increased in different degrees. Further, the increment of the number of iterations for ST-NN is 67, which is the largest among these four models. As a result, ST-NN has the largest computational time among all the neural network models under the second learning samples. It can be seen that strong tracking filtering needs more number of iterations to complete the learning process when the accuracy of learning samples is low. In contrast, the iterations of ST-NN, STSR-NN, and ISTSR-NN are not significantly different from those of the first group samples, indicating that square root filtering can improve the stability of iterative calculations in the learning process. Therefore, the computational time of ST-NN, STSR-NN, and ISTSR-NN are shorter than that of ST-NN with the second group of learning samples.

**Figure 6:**
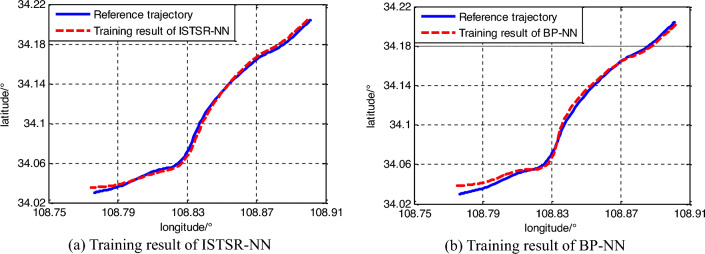
Training results of portion neural network models.

Figure 6 shows the training results and the desired. It can be seen that both the proposed NN model and BP-NN reached the training target, and then the output of intelligent navigation system is basically consistent with the reference trajectory. At the beginning and turning of the trajectory, there is a significant error in the output results of BP-NN, indicating that the proposed algorithm has higher training accuracy.

The above results and analysis demonstrate that in comparison with BP-NN, UKF-NN, SR-NN, ST-NN and STSR-NN, the proposed ISTSR-NN can suppress the influences of samples errors on the neural networks learning effectively, thus improving the adaptability and robustness of intelligent navigation system. Further, ISTSR-NN can also achieve the better computational performance in comparison with STSR-NN.

#### Prediction accuracy evaluation

When the learning process of neural networks were finished, the optimal network structure was obtained and applied to the error estimation of INS/GNSS intelligent integrated system.

**Figure 7:**
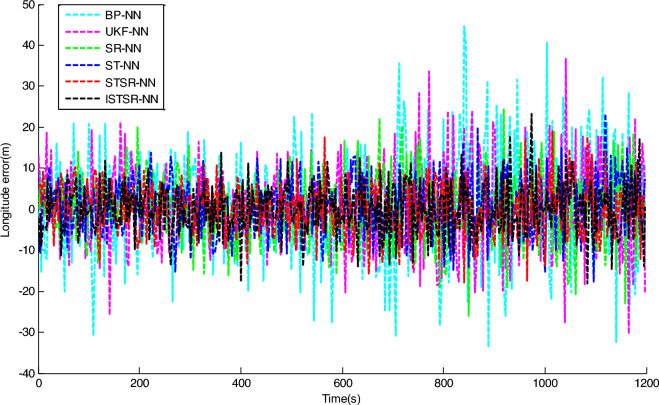
Longitude error by intelligent navigation system based on neural network models.

**Figure 8:**
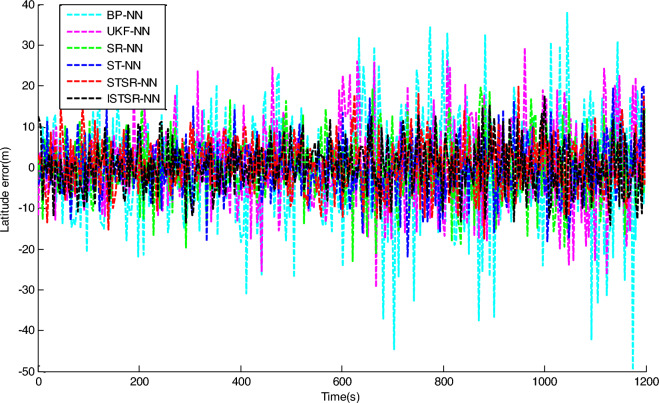
Latitude error by intelligent navigation system based on neural network models.

In order to comprehensively verify the effectiveness and accuracy of the method proposed in this article, two different learning samples are used to update the weight of the neural network during the learning process. Firstly, the actual output results of INS/GNSS combined filtering are used as the first group of learning samples for the neural networks. Secondly, the 10% random noise was added to the first group of samples and used as another group of learning samples for the neural networks. Finally, the two trained neural network models are applied to the intelligent navigation system to predict position errors when GPS is interrupted. The prediction results which are correspond to the time intervals (0 s, 600 s) and (601 s, 1200 s), respectively, are shown in Figs. 7 and 8.

It can be seen from Figs. 7 and 8 that the prediction accuracy of BP-NN is lowest. Its maximum position errors in east and north directions are 30.9 m and 30.8 m respectively during the time interval (0 s, 600 s), and the position errors further increase to 44.6 m and 49.7 m during the time interval (601 s, 1200 s). As a consequence, the intelligent navigation system based on BP-NN has poor positioning performance when GPS is interrupted. Compared with BP-NN, UKF-NN could effectively avoid the problem of local minimum in neural network learning by establishing state space equation to update the weight. So the maximum position errors of UKF-NN in east and north directions are reduced to 25.8 m and 25.6 m respectively during the time interval (0 s, 600 s). During the time interval (601 s, 1200 s), these data are also decreased to 36.8 m and 29.4 m respectively.

However, the amplitude of error fluctuation by UKF-NN is still significant. In contrast, the fluctuation amplitude of position error obtained by SR-NN is relatively small. During the time interval (0 s, 600 s), the maximum errors in east and north directions are 20 m and 21.2 m, respectively. Similarly, the maximum values of position error change to 25.9 m and 23 m during the time interval (601 s, 1200 s). Therefore, the positioning accuracy of intelligent navigation systems can be improved effectively while the GPS is interrupted by introducing the square root filter in the process of weight updating. Compared with SR-NN, the positioning accuracy of ST-NN has been further improved. During the time interval (0 s, 600 s), the maximum position errors in east and north directions are 15.3 m and 18.2 m respectively, while these data are 23.3 m and 22.0 m during the time interval (601 s, 1200 s). Based on the analysis above, the impact of sample noise on weight updating could be reduced effectively in the neural networks learning due to the tracking coefficient involved.

The position errors of STSR-NN and ISTSR-NN are close to that of ST-NN. Taking the latitude error as an example, their maximum position errors are 15.3 m and 13.7 m respectively during the time interval (0 s, 600 s), while these data are 19.7 m and 17.5 m during the time interval (601 s, 1200 s). When the satellite signal is invisible, the vibration amplitude of the position error curve of STSR-NN and ISTSR-NN are the smallest among the six neural network models.

Tables 3 and 4 show the parameters statistics results. During the time interval (601 s, 1200 s), both the RMSE of BP-NN and UKF-NN exceed 10 m due to the extra measurement error in the learning samples. In contrast, the square root is used to replace the error matrix for weight updating by SR-NN, which has better computational stability. Therefore, the NN model has better robustness, whose position RMSE are less than 10 m. Furthermore, ST-NN could effectively impede the perturbations caused by the measurement error in learning samples through its adaptive factor. Its position RMSE are around 7.76 m in longitude and 7.36 m in latitude, which are smaller than those of BP-NN, UKF-NN and SR-NN. Since STSR-NN can combine the advantages of SR-NN and ST-NN, its position RMSEs are around 6.79 m in longitude and 6.85 m in latitude, which are more small than that of SR-NN and ST-NN. Similarly, the position RMSE of ISTSR-NN are around 6.73 m in longitude and 6.55 m in latitude, which are very close to those of STSR-NN. Furthermore, the trend of changes in MAE and SI are very similar to that of RMSE.

**Table 3:** The statistics results of NN models for longitude errors.

	Time (s)	RMSE (m)	MAE (m)	SI	CC	U95	Reliability
BP-NN	0–600	10.21	8.21	7.21	0.92	1.42	75.88%
	601–1200	15.33	12.31	11.15	-0.85	2.11	72.61%
UKF-NN	0–600	7.65	6.00	4.64	0.91	1.06	76.95%
	601–1200	11.76	9.38	7.76	-0.82	1.63	74.82%
SR-NN	0–600	6.46	4.98	3.46	0.90	0.90	78.11%
	601–1200	9.27	7.33	5.27	-0.81	1.29	76.93%
ST-NN	0–600	5.87	4.88	2.86	0.91	0.82	79.02%
	601–1200	7.76	6.43	3.58	-0.82	1.07	77.23%
STSR-NN	0–600	5.33	4.34	2.31	0.89	0.74	83.70%
	601–1200	6.79	5.38	2.79	-0.83	0.94	82.07%
ISTSR-NN	0–600	5.20	4.23	2.20	0.92	0.72	85.19%
	601–1200	6.73	5.34	2.73	-0.82	0.93	83.95%

**Table 4:** The statistics results of NN models for latitude errors.

	Time (s)	RMSE (m)	MAE (m)	SI	CC	U95	Reliability
BP-NN	0–600	10.26	8.32	7.19	0.92	1.40	74.78%
	601–1200	16.02	12.71	12.01	0.78	2.22	72.79%
UKF-NN	0–600	7.54	5.80	4.54	0.93	1.04	77.12%
	601–1200	12.21	10.15	8.19	0.80	1.69	75.55%
SR-NN	0–600	6.31	5.01	3.30	0.93	0.88	78.81%
	601–1200	8.56	6.79	4.56	0.79	1.18	77.32%
ST-NN	0–600	5.52	4.41	2.52	0.94	0.77	79.00%
	601–1200	7.36	5.74	3.35	0.79	1.01	77.63%
STSR-NN	0–600	5.42	4.27	2.39	0.93	0.75	83.54%
	601–1200	6.85	5.57	2.84	0.80	0.95	81.90%
ISTSR-NN	0–600	5.13	4.00	2.13	0.94	0.71	85.94%
	601–1200	6.55	5.31	2.54	0.81	0.91	83.66%

On the other hand, it can be seen that the increment of position errors of STSR-NN and ISTSR-NN between the two time intervals are the smallest. The increment of position errors of SR-NN and ST-NN are more larger than that of STSR-NN and ISTSR-NN, while the increment of position errors of BP-NN and UKF-NN is the largest. Therefore, the intelligent navigation systems based on BP-NN and UKF-NN heavily rely on the accuracy of learning samples. Once the learning samples are not accurate enough, the positioning performance is very poor when satellite signals are interrupted. In contrast, STSR-NN and ISTSR-NN introduce both square root filtering and strong tracking filtering during the process of weight updating, making the iterative calculation of the network more stable. Meanwhile, STSR-NN and ISTSR-NN adjust the filtering gain by tracking coefficients, making the trained neural networks more robust and adaptive. Therefore, intelligent navigation systems based on STSR-NN and ISTSR-NN can achieve better positioning accuracy when the satellite is interrupted.

It should also be noted that for the time intervals (0 s, 600 s) without involving the measurement error in the learning samples, the positioning errors of BP-NN is significantly larger than UKF-NN and SR-NN due to the problem of local minimum in BP algorithm, while SR-NN has a higher positioning accuracy than UKF-NN because of the Cholesky and QR decomposition involved in the recursive operation for the stability of numerical calculation. Moreover, the positioning accuracy of ISTSR-NN is slightly higher than that of STSR-NN. This is because STSR-NN are suboptimal when the tracking coefficient was calculated, while the ISTSR-NN is optimal. Accordingly, STSR-NN follows ISTSR-NN’s positioning results during this time intervals, leading to a similar level of positioning accuracy.

Therefore, by comparing the statistics results of each NN models within the different time periods together, it is evident that the proposed ISTSR-NN has the higher positioning accuracy and reliability than the other fiver NN models for the entire simulation time.

### Experiments and analysis

A practical experiment for vehicle positioning with intelligent navigation system was conducted to further evaluation the performance of the proposed ISTSR-NN. The comparison analysis with BP-NN, UKF-NN, SR-NN, ST-NN and STSR-NN were also carried out based on this practical experiment.

**Figure 9:**
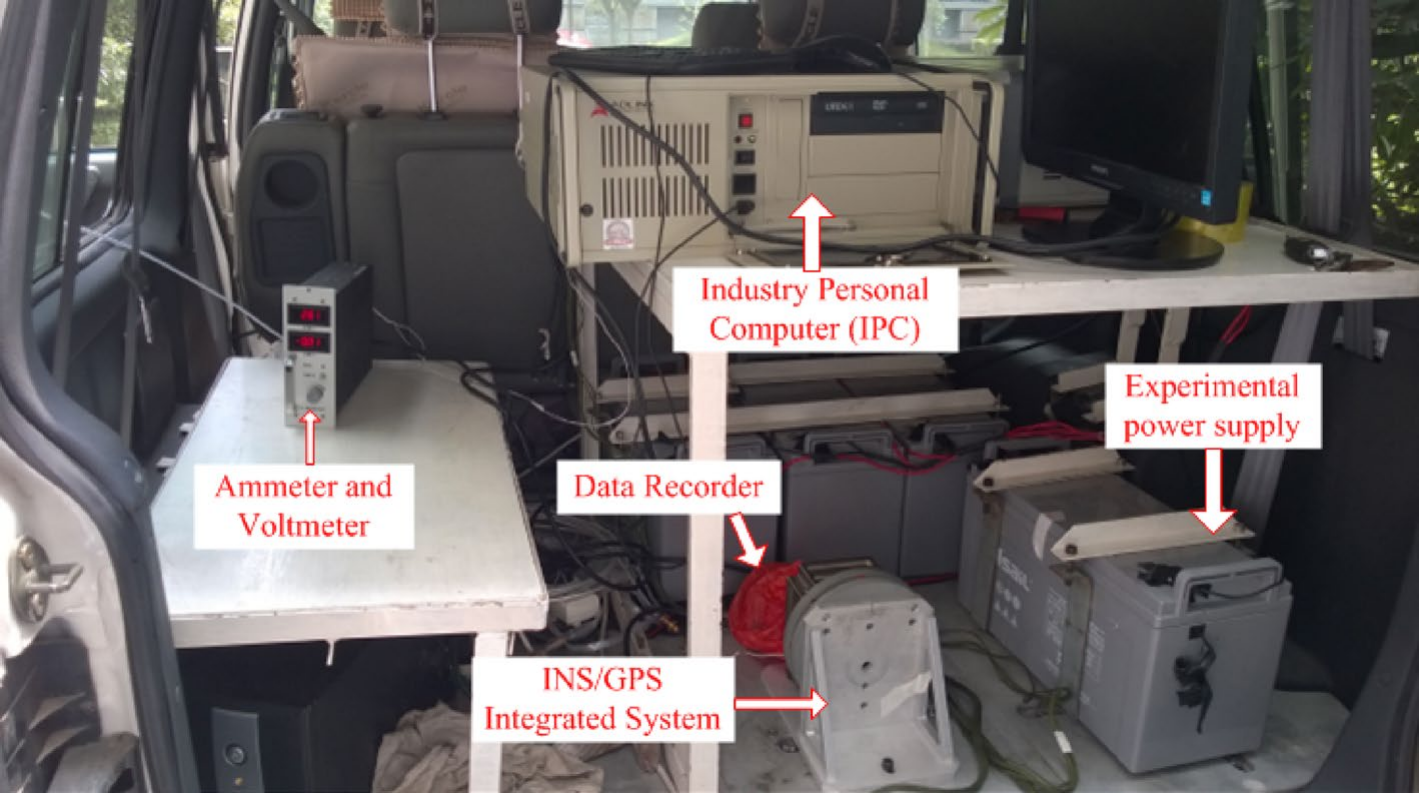
Experimental vehicle and its carry experimental instruments.

The test vehicle and internal test instruments are shown in Fig. 9. The vehicle uses an INS/GPS integration system for navigation. This navigation system includes a NV-300 IMU and a JAVAD Lexon-GGD112T GPS receiver. The main parameters of the above two devices are listed in Tables 5 and 6. Moreover, another JAVAD Lexon-GGD112T GPS receiver, which was placed at a local reference station (around 1 km from the initial UAV position), was used along with the one mounted on the vehicle to provide the differential GPS (DGPS) data. The maximal distance between the vehicle and local reference station was less than 60 km to achieve the position accuracy of less than 0.1 m from the DGPS via post difference processing. The DGPS data were used as the reference values to evaluate the positioning error of the intelligence navigation system.

**Table 5:** Noise parameters of the NV-300 IMU.

Noise Parameters		Values
Gyro	Constant drift	0.06°/h
	Random walk coefficient	0.01°/$\sqrt h$
Accelerometer	Zero-bias	5 × 10^-3^ g
	Random walk coefficient	1 × 10^-4^ g∙$\sqrt s$

**Table 6:** Main parameters of JAVAD Lexon-GGD 112 T GPS receiver.

Feature Parameters	Values
Satellite signals	GPS L1/L2
Horizontal position error (RMS)	5 m
Altitude error (RMS)	8 m
Velocity error (RMS)	0.05 m/s
Data update rate	1 Hz

#### Long-time interruption tests

**Figure 10:**
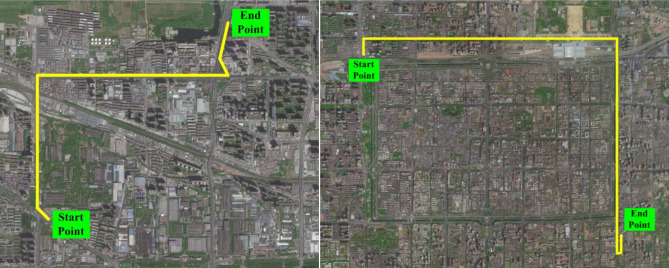
The vehicle trajectory for the experimental case.

The vehicle positioning tests were carried out in Xi’an, China. The vehicle trajectories are depicted in Fig. 10. The trajectory includes straight, turn round, turn around, etc. The experimental data obtained from these two trajectories are used to learning and prediction of the intelligent navigation system respectively. When training the intelligent navigation system by the data of learning trajectory, the GPS signals are in a normal working state. On the other hand, when using the data of predicted trajectory to verify the estimation performance of intelligent navigation systems, the GPS signals are in an interrupted state. The information of trajectories are shown in Table 7.

**Table 7:** Information of experimental path.

Purpose	General information	Starting coordinates	Ending coordinates	GPS status
Learning	Duration: 840 s Distance: 6.2 km	East longitude 108.880°, North latitude 34.279°	East longitude 108.898°, North latitude 34.292°	Normal
Prediction	Duration: 1200 s Distance: 9.5 km	East longitude 108.929°, North latitude 34.283°	East longitude 108.979°, North latitude 34.256°	Interrupt

**Figure 11:**
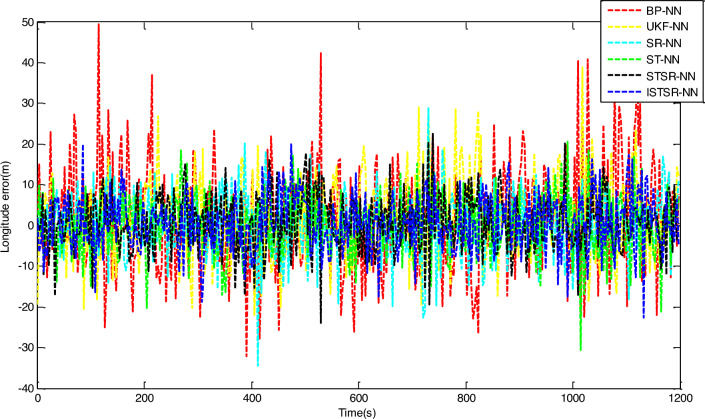
Longitude error for practical experiment based on neural network models.

**Figure 12:**
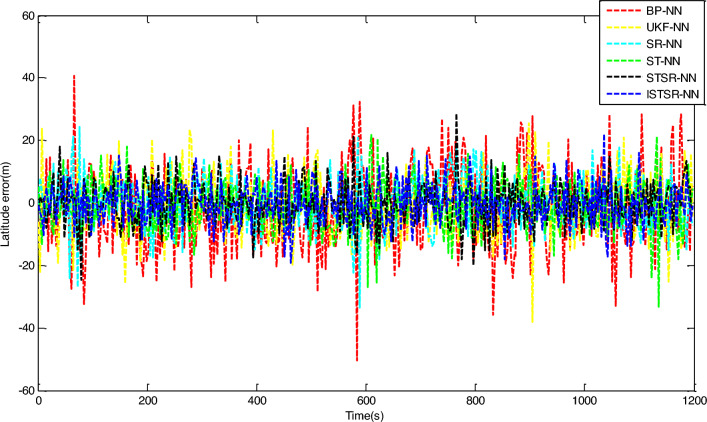
Latitude error for practical experiment based on neural network models.

The longitude and latitude error of the vehicle by BP-NN, UKF-NN, SR-NN, ST-NN, STSR-NN and ISTSR-NN are shown in Figs. 11 and 12, respectively. Moreover, Tables 8 and 9 show the detailed parameter statistics results. During the vehicle positioning experimental, the intelligent navigation system inevitably involved measurement errors in the learning samples due to the vehicle maneuvering and the disturbances of the external environment in the urban area.

**Table 8:** The statistics results of NN models for longitude errors.

	RMSE (m)	MAE (m)	SI	U95	CC	Reliability
BP-NN	13.03	10.07	8.89	1.27	0.93	74.26%
UKF-NN	9.69	7.61	6.61	0.95	0.93	76.10%
SR-NN	7.54	5.75	4.53	0.74	0.92	77.95%
ST-NN	7.15	5.60	3.15	0.70	0.93	78.07%
STSR-NN	6.90	5.37	2.86	0.68	0.91	81.64%
ISTSR-NN	6.78	5.30	2.76	0.67	0.92	83.39%

**Table 9:** The statistics results of NN models for latitude errors.

	RMSE (m)	MAE (m)	SI	U95	CC	Reliability
BP-NN	13.12	10.61	8.98	1.26	0.95	73.92%
UKF-NN	9.31	7.48	6.28	0.91	0.93	76.23%
SR-NN	8.07	6.37	4.07	0.79	0.95	77.45%
ST-NN	7.59	5.94	3.54	0.73	0.95	78.18%
STSR-NN	6.70	5.15	2.70	0.66	0.94	81.84%
ISTSR-NN	6.32	4.93	2.31	0.62	0.93	83.71%

The solution of BP-NN are significantly affected by measurement errors in the learning samples, resulting in large oscillations in the positioning error curve. The RMSE of BP-NN in longitude and latitude are 13.03 m and 13.12 m respectively. The RMSE of UKF-NN are smaller than those of BP-NN. This is because UKF-NN uses nonlinear filtering to train the neural network, which can effectively avoid defects such as falling into local minima during training. However, the MAE of UKF-NN in longitude and latitude are 7.61 m and 7.48 m, while the SI of UKF-NN in longitude and latitude are 6.61 and 6.28. In contrast, the MAE and SI of both SR-NN and ST-NN are smaller than those of the UKF-NN. This is mainly because SR-NN and ST-NN improve the computational stability and adaptability by embedding the QR decomposition and strong tracking factor during the neural network learning, respectively.

Furthermore, STSR-NN improves both SR-NN and ST-NN, and its RMSE are 6.90 m and 6.70 m. This is because both QR decomposition and strong tracking factor are involved in STSR-NN, thus the higher positioning accuracy and robustness could be achieved. Finally, ISTSR-NN’s RMSE are 6.78 m and 6.32 m, while the MAE are 5.30 m and 4.93 m, all of which are the smallest within the six algorithms. This is mainly because the proposed ISTSR-NN in this article improves the calculation method of strong tracking factors, thus further improving the accuracy of neural network training and the prediction accuracy of intelligent navigation systems. Therefore, ISTSR-NN has a better both adaptability and robustness than other five algorithms. At last, as shown in Tables 8 and 9, ISTSR-NN has reached the best results in reliability, better than other five NN methods.

The above experimental results indicate that ISTSR-NN has both adaptability and robustness against measurement errors in the training samples, leading to higher positioning accuracy and reliability than BP-NN, UKF-NN, SR-NN, ST-NN and STSR-NN for intelligent navigation system. In addition, the training efficiency and accuracy of these six algorithms for intelligent navigation system are similar to those of the simulation test, which can prove that the improved calculation method for strong tracking factor has obvious positive effect on improving the real-time performance of the learning algorithm. Therefore, the discussion on training performance of the algorithms will not be repeated here.

#### Short-time interruption tests

**Figure 13:**
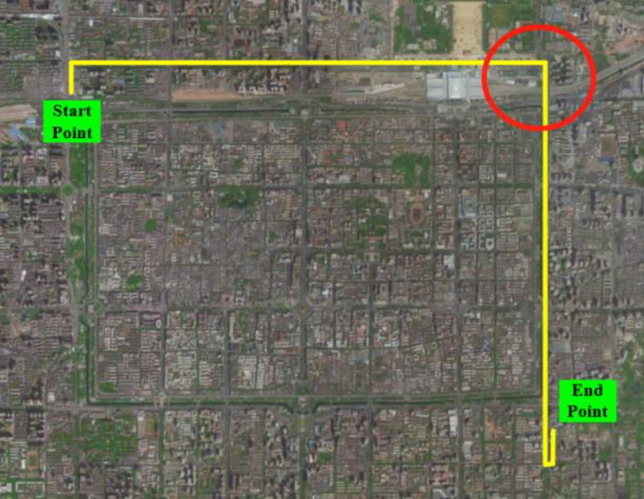
The vehicle trajectory for the experimental case.

Short term signal interruptions of tens of seconds to a few minutes often occur in narrow areas with dense high-rise buildings and areas with poor observation environments inside buildings in cities. To verify the predictive performance of ISTSR-NN in this condition, this section establishes a short-term GPS outage in the predicted trajectory of Fig. 10. The interruption area is shown in Fig. 13 and the interruption time is set as 120 s.

For short-term interruption conditions, a window based weight update strategy is used to update the network parameters, with a window size set to 120 s. When the GPS signal is available, the intelligent navigation system operates in training mode. When the satellite signal is interrupted, the intelligent navigation system switches to prediction mode. The advantage of this setting is that the NN model can use the last updated network parameters for prediction.

**Figure 14:**
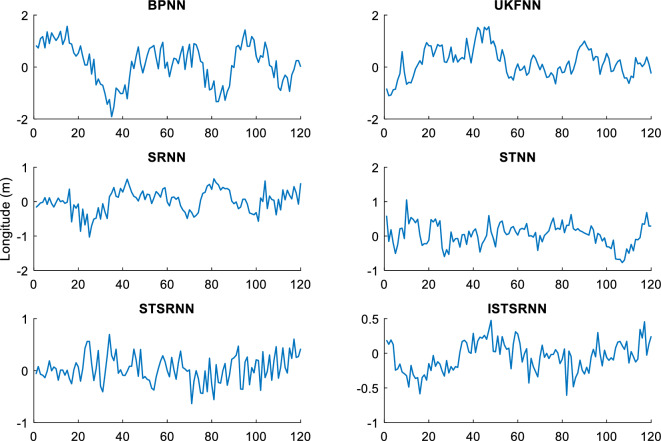
Longitude error results during 120 s interruption.

**Figure 15:**
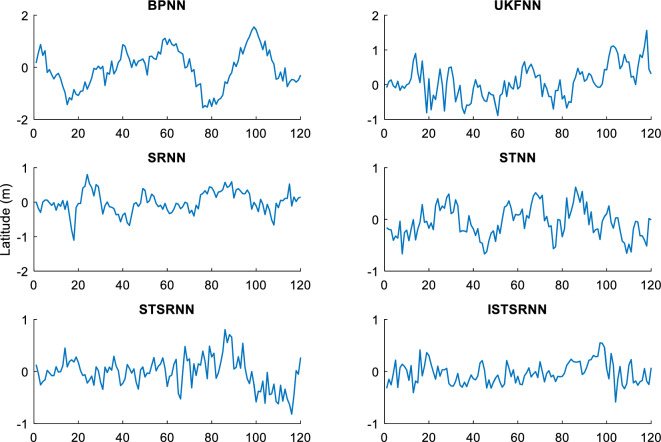
Latitude error results during 120 s interruption.

The short-term position prediction results for 120 s are shown in Figs. 14 and 15. According to the simulation results, the RMSE of BP-NN in longitude and latitude are 0.78 m and 0.74 m respectively. The RMSE of UKF-NN are smaller than those of BP-NN, which are 0.59 m and 0.48 m. In addition, the RMSE of both SR-NN and ST-NN are smaller than those of the UKF-NN, and the prediction results of SR-NN and ST-NN are very close. Finally, STSR-NN and ISTSR-NN have the highest prediction accuracy, with the RMSE of the latitude and longitude below 0.3 m. This results indicate that the proposed training algorithm can still effectively improve the navigation accuracy of intelligent systems and enhance the robustness of NN in short-term interruption conditions.

Finally, it should be pointed out that if satellite signals are sometimes interrupted and sometimes visible in practical applications, the intelligent navigation system needs to constantly switch between training mode and prediction mode. Therefore, in order to balance the requirements of training accuracy and computational complexity more better, ISTSR-NN is the best choice for intelligent navigation systems.

## Discussion

This paper presents an ISTSR-NN learning algorithm to improve the adaptability and numerical stability for INS/GNSS intelligent integrated system. The contributions of this paper are as follows.

From the experiment tests, it can be seen that the proposed ISTSR-NN learning algorithm can effectively reduce the impact of the server samples noise, which was produced by the dynamic environment, improve the learning effective of the neural network model, and then improve the prediction accuracy of navigation parameters, which verifies the feasibility of the proposed method.

Compared with the BP-NN and UKF-NN, the fluctuation amplitude of position error which were processed by ISTSR-NN is obviously reduced. It is proved that the proposed algorithm has better computational stability. On the other hand, in the face of the sudden change of noise intensity, the proposed algorithm has better positioning performance, which shows that it can effectively improve the adaptive ability of the mutation state.

The improved calculation method of strong tracking coefficient can effectively reduce the calculation complexity and learning time-consuming so that the algorithm can effectively improve the real-time performance of intelligent integrated system.

The future research work will focus on the improvement of intelligent integrated system. By designing reasonable input and output variables of neural network, the velocity and attitude errors of space carrier could be predicted.

## Data Availability

The datasets generated and analyzed during the current study are not publicly available due to the secret restrictions with data providers but are available from the corresponding author on reasonable request.
